# Ergogenic Effects of β-Alanine and Carnosine: Proposed Future Research to Quantify Their Efficacy

**DOI:** 10.3390/nu4070585

**Published:** 2012-06-26

**Authors:** John Caruso, Jessica Charles, Kayla Unruh, Rachel Giebel, Lexis Learmonth, William Potter

**Affiliations:** 1 Exercise & Sports Science Program, The University of Tulsa, Tulsa, OK 74104, USA; Email: jessica-charles@utulsa.edu (J.C.); kayla-unruh@utulsa.edu (K.U.); rachel-giebel@utulsa.edu (R.G.); lexis-learmonth@utulsa.edu (L.L.); 2 Department of Chemistry & Biochemistry, The University of Tulsa, Tulsa, OK 74104, USA; Email: william-potter@utulsa.edu

**Keywords:** carnosine, dietary supplement, amino acid, paraesthesia

## Abstract

β-alanine is an amino acid that, when combined with histidine, forms the dipeptide carnosine within skeletal muscle. Carnosine and β-alanine each have multiple purposes within the human body; this review focuses on their roles as ergogenic aids to exercise performance and suggests how to best quantify the former’s merits as a buffer. Carnosine normally makes a small contribution to a cell’s total buffer capacity; yet β-alanine supplementation raises intracellular carnosine concentrations that in turn improve a muscle’s ability to buffer protons. Numerous studies assessed the impact of oral β-alanine intake on muscle carnosine levels and exercise performance. β-alanine may best act as an ergogenic aid when metabolic acidosis is the primary factor for compromised exercise performance. Blood lactate kinetics, whereby the concentration of the metabolite is measured as it enters and leaves the vasculature over time, affords the best opportunity to assess the merits of β-alanine supplementation’s ergogenic effect. Optimal β-alanine dosages have not been determined for persons of different ages, genders and nutritional/health conditions. Doses as high as 6.4 g day^−1^, for ten weeks have been administered to healthy subjects. Paraesthesia is to date the only side effect from oral β-alanine ingestion. The severity and duration of paraesthesia episodes are dose-dependent. It may be unwise for persons with a history of paraesthesia to ingest β-alanine. As for any supplement, caution should be exercised with β-alanine supplementation.

## 1. Introduction

In the never-ending quest for improved athletic performance, persons interested in greater exercise prowess and physical appearance have ingested a variety of substances touted to act as ergogenic aids, sometimes to the point of an increased risk to their own health. Among the newer dietary supplements popular with athletes and fitness enthusiasts is β-alanine, an amino acid that has a myriad of purported health and exercise benefits. Recent reviews on this topic [1,2,3,4,5] concluded their examinations with calls for continued research. While these prior reviews [1,2,3,4,5] provided valuable information, perhaps the full extent of β-alanine’s prowess as an ergogenic aid has likely yet to be accurately and objectively quantified. For instance there appears to be a very strong disconnect between the proposed way oral β-alanine may act as an ergogenic aid, and the type of outcome measures and studies used to assess its efficacy. Adding to the confusion, a recent meta-analysis shows a heightened exercise capacity, yet no significant improvement in performance, from oral β-alanine supplementation [3]. Meta-analysis results included a median β-alanine intake of 179 g per intervention led to a median improvement of less than 3% [3]. Included in the meta-analysis was a study [6] that appears to be an outlier based on its large increase in median effect size. 

The disparity in results from these recent reviews [1,2,3,4,5,6] clearly indicate the types of studies and exercise bouts previously employed have not allowed oral β-alanine supplementation to be optimally examined and utilized for its ergogenic properties. It is clear from examinations of cellular biochemistry that exercise which evokes intracellular H^+^ accrual may benefit most from oral β-alanine intake. Yet some reviews [2,3,4] offered limited information on exercise-induced cellular conditions (metabolic acidosis) that led to increased β-alanine and carnosine research, as well as little quantitative evidence of the latter’s prowess to continue to act as a potent buffer in the face of successively larger intramuscular pH decrements. Far from a mere recitation and review of prior study outcomes, this paper offers insights as to how β-alanine may best be used, as well as how its ergogenic properties can be objectively and accurately quantified through continued exercise-based research. In order to provide more information on β-alanine, this review will also describe its metabolism, roles within the body, ergogenic effects and discuss side effects that may result from its ingestion.

## 2. Experimental Section

### 2.1. β-Alanine

**Figure 1:**
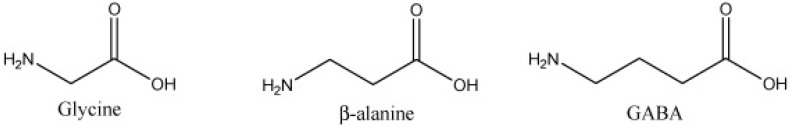
The structural similarities of glycine, β-alanine and GABA.

β-Alanine is a non-proteinogenic amino acid that may be produced within the human body [2]. Its synthesis within the body occurs from the breakdown of pyrimidines, decarboxylation by gut microbes of L-aspartate and the transamination with 3-oxopropanate by L-aspartate [7,8,9,10]. Endogenous β-alanine synthesis occurs within the liver from the irreversible degradation of thymine, cytosine and uracil [2]. Once synthesized it is transported to muscle cells where it crosses the sarcolemma via a Na^+^- and Cl^−^-dependent process [1]. β-alanine uptake is comparable among different skeletal muscle fiber types [1]. Intracellular β-alanine uptake relies on the same co-transporter for delivery of substances that possess a similar structure (Figure 1), such as glycine, taurine and GABA [1]. Thus it is possible β-alanine supplementation impairs, via competitive inhibition, uptake of these other substances [1,11]. Yet β-alanine dosages that impair intracellular taurine levels far exceed those given to humans [1], thus concerns about compromised intramuscular taurine concentrations from β-alanine administration are unfounded [11]. β-alanine has several functions; within the nervous system it may act as a neurotransmitter or neuromodulator and has hippocampal binding sites on NMDA, GABA-A, GABA-C and glycine receptors to aid the learning of new information [9].

**Figure 2:**
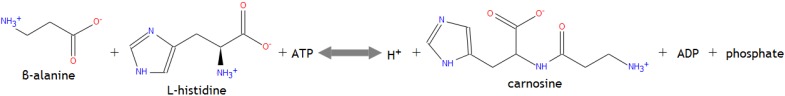
Intramuscular carnosine formation.

Within muscle cells β-alanine may combine with the amino acid histidine to form the dipeptide molecule carnosine. Since histidine is more abundant in muscle cells than β-alanine, the latter is considered the rate limiting amino acid to carnosine formation [11]. The dipepetide is created through an ATP-dependent reaction shown in Figure 2 [10].

Catalyzed by carnosine synthetase, its formation occurs primarily in muscle cells, as the dipeptide’s levels in the bloodstream are negligible due to the catalytic actions of serum carnosinase [2,10]. Yet intramuscular transport pathways for carnosine, via oligopeptide transporters and ion-dependent mechanisms, bypass the degradative effects of carnosinase [2]. The p*K*_a_ of histidine’s imidazole ring, commonly known as the acid dissociation constant, is 6.1. Yet when histidine binds to β-alanine the p*K*_a_ of the imidazole ring rises to 6.83 and has a major impact on H^+^ buffering [2]. Despite its numerous functions, β-alanine’s ergogenic effects may be maximized when it is incorporated into carnosine molecules. Thus carnosine’s actions also warrant inquiry.

### 2.2. Carnosine

Carnosine synthesis is regulated by (1) the rate and magnitude of β-alanine uptake within muscle fibers, (2) serum carnosine synthase activity and, in the absence of sufficient β-alanine within the diet, (3) hepatic synthesis of the amino acid and its transport to skeletal muscle [11,12,13]. Intracellular β-alanine levels may have the greatest impact on subsequent carnosine synthesis [12]. Yet some believe the uptake of the amino acid into muscle acts as the rate-limiting factor [11]. Carnosine may be delivered across cell membranes through H^+^-dependent oligopeptide transporters [14,15,16]. Intracellular carnosine metabolism may be a contractile-dependent process, as its transport to muscles rises while they are under tension [2]. Normal intramuscular carnosine levels of 20–30 mmol·kg^−1^ dry weight (5–8 mmol L^−1^) are like those for ATP, carnitine and taurine [2]. Across species, greater anaerobic metabolism evokes higher intramuscular carnosine [2,17].

Carnosine is a stable molecule; its intramuscular concentrations usually show only minor changes over time [2,18]. High initial intramuscular carnosine levels do not apparently limit a cell’s ability to accrue further increases [19]. Carnosine content by muscle fiber types show the following hierarchical order: I < IIa < IIx [9,11,13]. Intracellular levels are higher in men than women, and decline with age in both genders as within glycolytic muscle fibers there is 47% less carnosine elderly (70.4 ± 5.0 years) *versus* young (23.8 ± 4.6 years) subjects [2,6,20]. Such differences may be related to testosterone, as a correlation between carnosine and the hormone exist; yet a direct “cause-and-effect” has not been established [2,21,22].

Like β-alanine, carnosine has multiple functions. They include inhibition of lipid and protein oxidation, ATPase activation, macrophage regulation, cell membrane protection, divalent cation chelation, protection of numerous proteins against glycation, as an anti-aging agent and a regulator of cross-bridge formation via changes in Ca^2^^+^ sensitivity [2,10,23,24,25,26,27]. Its levels within the brain (0.1 mmol/L) may limit the risk of neurotoxicity from copper- and zinc-based compounds [28]. Carnosine may also act as a neurotransmitter, but it is likely of most interest to athletes and fitness enthusiasts for its ability to abate exercise-induced fatigue [5,10,24,25,29]. Study results on carnosine’s ergogenic properties are mixed, thus it is important to elucidate how it best aids performance. Therefore to understand how carnosine best abates fatigue, it is important to describe intracellular conditions that impede exercise performance.

#### Carnosine and Exercise-Induced Fatigue

**Figure 3:**
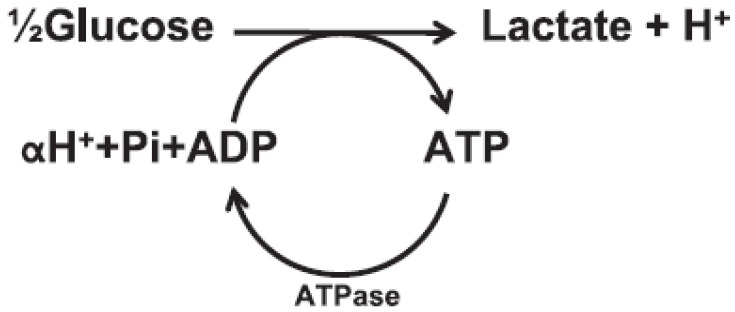
Anaerobic glycolysis and ATP hydrolysis schematic; carnosine buffers H^+^ increases.

As persons exercise to voluntary failure, multiple mechanisms (psychological, neurological, metabolic, *etc.*) may undermine performance. Since carnosine is an intramuscular compound, it is important to examine conditions within cells as exercise progresses to the point of fatigue. In the quest for sufficient ATP, perhaps the greatest exercise-induced intracellular change is the rapid accrual of lactate and H^+^ that typifies metabolic acidosis (Figure 3). Lactate and acid metabolites accrue commensurate to the degree by which intracellular aerobic capacity is exceeded, such as when anaerobic glycolysis serves as the primary ATP resynthesis pathway [30]. At maximal exercise rates, such bouts last from 15 to 240 s [4,30]. Intramuscular glycolytic rates may rise 1000-fold as persons go from rest to supramaximal exercise [31].

With supramaximal exercise, intracellular ATP declines up to 40% and causes a near complete depletion of phosphocreatine as well as elevations in lactate and H^+^ [32]. Intramuscular pH, from resting values of ~7.1, may decline from supramaximal activity to less than 6.5, which represents a four-fold rise in H^+^ and an intracellular increase of up to 54 mmol·kg^−1^ [33,34,35]. When such increases are added to other reactions that raise the total proton load intracellular H^+ ^levels exceed 100 mmol·kg^−1^ [36]. Concomitant lactate increases also occur with higher glycolytic rates; intramuscular and plasma levels for the metabolite rise by up to 40 and 25 mmol·L^−1^ [34,36,37,38]. A strong linear relationship exists between muscle pH decrements and intracellular lactate and pyruvate values [39]. Thus examinations of blood lactate changes over time, which quantify the rate of the metabolite’s entry into and removal from the vasculature, foretell concurrent intracellular H^+^ changes in response to, and recovery from, exercise.

Upon cessation of exercise, force output and intracelluar pH restoration is rapid, yet the former recovers at a faster rate [32,33]. For each measure, recovery is slower than their rate of loss incurred from supramaximal exercise [32,33]. Adverse intracellular changes from H^+^ accrual include phosphofructokinase inhibition, impaired phosphocreatine resynthesis, slower cross bridge transitions from low- to high-force states, losses in maximal shortening velocity, lowered glycolytic rates, competitive inhibition with Ca^2^^+^ at the troponin C subunit, and delayed Ca^2^^+^ reuptake by the sarcoplasmic reticulum [3,30,40]. Such changes compromise exercise and recovery rates, which is a concern if successive bouts of supramaximal activity are to occur [32]. If contractions proceed at high force outputs, which are common to supramaximal exercise, high H^+^ levels also impair intramuscular Ca^2^^+^ release and compromise Ca^2^^+^-ATPase activity [41].

To cope with such conditions cells utilize multiple mechanisms to remove lactate and H^+^, as well as buffers to mitigate the effects of metabolic acidosis. Lactate and H^+^ efflux is facilitated by monocarboxylate transporters such as MCT1 and MCT4 [40]. Unlike carnosine, which appears to have no ceiling with respect to its intramuscular concentration [18], other intracellular buffers such as proteins, bicarbonates, citrates and NAD^+^-dependent redox reactions, are under tight homeostatic control [42]. In untrained persons, carnosine typically adds a mere 7%–10% to their intracellular buffering capacity and neutralizes 2.4–10.1 mmol H^+^·kg^−1^ dry mass as intramuscular pH declines [35,43]. Yet during exercise-induced metabolic acidosis carnosine is particularly effective as an H^+^ acceptor to due to its imidazole ring and a concomitant shift in its p*K*_a_ to 6.83, which is rare for intracellular buffers and vital to maintain pH.

Unlike most buffers, carnosine is potent over a broad pH range in type I and II muscle fibers from the rapid and substantial accrual of intracellular H^+^ and lactate. Carnosine levels range from 4 to 7 and 9 to 13 mM within type I and II fibers respectively [44,45]. Since buffer capacity (β) is a quantitative index of the resistance of a buffer solution to change pH upon added H^+^, its formula is as follows:

β = (Δn)/(ΔpH)     (1)

where Δn represents the incremental additions of proton equivalents to a buffered system and ΔpH is the observed pH changes. This equation is commonly known as the buffer derivative, in which pH (*i.e.*, −log_10_[H^+^]) is expressed as [H^+^] and where the β in the presence of a neutralizing agent with an acid dissociation constant represented by *K*_a_, is defined as:


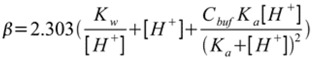
     (2)

It is important to note, for near neutral pH’s, the first (K_w_/[H^+^]) and second ([H^+^]) terms do not contribute significantly to carnosine’s prowess as a buffer. β can be calculated at pH = 7.1 for a lower limit of 4 × 10^−3^ M carnosine in type I fibers to have a buffer capacity of 9.1 × 10^–4^ [42]. At pH = 7.1 for carnosine measured at the higher range found in type II fibers, *i.e.*, at 13 × 10^−3^ M, the buffer capacity rises to 2.95 × 10^−3^, approximately a 3.2-fold increase [42]. At a pH of 6.5, the 4 mM carnosine only slightly changes to 8.7 × 10^−4^, and for the 13 mM carnosine to 2.8 × 10^−3^. Thus as pH declines from 7.1 to 6.5 in exercising muscle, carnosine’s buffer capacity is well preserved in type I and II fibers. Not mentioned in the prior reviews [1,2,3,4,5], and unlike buffers and enzymes that only operate within a limited pH range, carnosine’s ability to absorb protons is well maintained over a substantial pH range and makes it a unique and valuable molecule. Thus this paper will now elaborate on the efficacy of prior interventions to raise intracellular carnosine levels.

### 2.3. The Impact of Exercise and Dietary Interventions on Carnosine Concentrations

While carnosine normally comprises 7%–10% of a muscle’s buffer capacity, its levels may be raised through exercise. Elite athletes typically possess the most carnosine, which is likely a training adaptation [1,2,21,22]. High intramuscular carnosine levels were seen in bodybuilders [22]. However that data may be deemed as suspect since subject’s diets were not controlled and they ingested anabolic steroids; thus carnosine content may be linked to testosterone [22]. Yet if exercise raises carnosine levels, it does so slowly. Short-term training studies that quantified carnosine changes usually show small perturbations [1,2]. To date only one study showed significant carnosine gains, which occurred after eight weeks of cycle ergometer sprint exercise by untrained subjects [46].

Since intracellular carnosine accrual from exercise is arduous at best, research then assessed the extent whereby oral β-alanine raised intramuscular levels of the dipeptide. Single oral β-alanine doses in men, of either 10, 20 or 40 mg·kg^−1^ body mass, led to peak plasma values (mean ± SE) of 47 ± 13, 374 ± 68 and 833 ± 43 µM respectively, with pre-treatment levels restored two hours post-ingestion [25]. Two weeks of β-alanine, at a dose of 10 mg·kg^−1^ body mass three times daily, saw plasma levels for the amino acid return to baseline before the next dose was given [25]. Yet four week doses of 3.2 and 6.4 g·day^−1^ saw intramuscular carnosine rise 42 and 64% over pre-treatment values and saw its contribution to muscle buffer capacity rise 12.6 and 18% respectively [25,36,44].

Other trials noted elevated carnosine from oral β-alanine. At rates of 4–6 g·day^−1^, four weeks of β-alanine raised intramuscular carnosine ~60% [47,48,49]. A 56-day β-alanine treatment at lower doses (2–4 g·day^−1^) elicited slightly less (50%) carnosine accrual [18,25,49,50]. In a study that assessed carnosine levels by muscle group in order to yield information on changes by fiber type, men received oral β-alanine at rates of up to 4.8 g·day^−1^ for 5–6 weeks [18]. Results showed significant carnosine gains to the soleus (39%), tibialis anterior (27%) and medial gastrocnemius (23%) [18]. It was implied that while higher carnosine contents are seen in glycolytic fibers, the treatment evoked greater relative gains in the oxidative soleus muscle [18]. Longer interventions at higher (4.8–6.4 g·day^−1^) doses raised intramuscular carnosine by 60 and 80% after four and ten weeks respectively [5,24,50,51]. To date, the greatest intramuscular increases occurred after 12 weeks of β-alanine administration at a rate of 3.2 g·day^−1^ in elderly subjects, a group thought to have naturally lower levels of the dipeptide [2,6,52]. Such changes presumably provide an ergogenic effect.

#### Oral β-alanine as an Ergogenic Aid to Exercise Performance

Consequently research examined the role of oral β-alanine supplementation on exercise performance; which has yielded mixed results. Many β-alanine trials simply examined for the presence of an ergogenic effect [44,46,47,48,49], and could not accurately quantify the full merits of this dietary supplement. One such study randomized college-age men to a placebo or β-alanine treatment with no crossover [49]. β-alanine-dosed subjects ingested an average of 5.2 g·day^−1^ for four weeks; a subset continued the intervention for ten weeks and consumed an average of 5.9 g·day^−1^. Before and after each dosing regimen, subjects performed a graded cycle ergometry test to the point of volitional fatigue. While placebo administration did not improve performance, β-alanine-dosed subjects produced significantly more work after four (+7.3%) and ten (+8.6%) weeks of supplementation as compared to baseline values [49]. Yet a far different result was seen from a time trial cycling test, which is a much more sport-specific outcome measure [53]. The time trial study administered concurrent β-alanine (65 mg·kg^–1^) and NaHCO_3_ (300 mg·kg^−1^) dosages to elite cyclists; presumably they would act as intra- and extra-cellular buffers respectively, and their combined actions would yield greater ergogenic effects [53]. Yet after a 28-day capsule dosing intervention, results showed placebo-NaHCO_3_ and β-alanine-NaHCO_3 _time trials improved performance to similar extents [53]. It was implied β-alanine did not improve performance beyond that conferred by NaHCO_3_ ingestion [53].

Other studies yielded a myriad of results which appear to be a function of the type of outcome measure examined. The acute effects of a dietary supplement, which included a 4 g dose of β-alanine, were examined for improvements in performance to a series of exercise tests [54]. Twelve recreationally-trained men ingested either the supplement or a placebo. Twenty minutes after ingestion, subjects performed the exercise tests. One week later they received the opposite treatment and performed identical ingestion and test procedures. The supplement produced significant improvements in acute resistive exercise performance at submaxmial loads, perceived energy and physical agility *versus* placebo administration [54]. It was suggested the supplement delays fatigue produced by strenuous exercise. Yet given the limited evidence that β-alanine offers an ergogenic effect from acute ingestion because intracellular carnosine accrual occurs gradually over time [1,2], performance improvements from the supplement likely resulted from another ingredient in its proprietary blend.

Most other studies examined exercise performance changes from chronic β-alanine intake. β-Alanine (4 g·day^−1^) or a placebo was given to college wrestlers and football players for eight weeks, with no crossover, to compare changes in exercise performance [55]. Before and after the intervention, subjects engaged in numerous tests related to anaerobic exercise performance. During the intervention both groups continued their customary exercise programs. A comparison of pre- and post-test measurements included non-significant inter-group differences. Yet some test measures showed a trend towards improvement from oral β-alanine intake [55]. It was suggested β-alanine may enhance performance and lean body mass accrual from improvements to the quality of an athlete’s exercise program [55]. However elderly subjects randomized to 12 weeks of β-alanine (3.2 g·day^−1^) or placebo administration with no crossover noted the former treatment yielded significant pre-post improvements in incremental exercise performance [52]. Yet another study randomized subjects to a 28-day β-alanine (6 g·day^−1^) or placebo treatment with no crossover to examine responses to an incremental running protocol [56]. Before and after the intervention subjects performed the protocol on a treadmill, which entailed successively more difficult stages until they reached volitional exhaustion. Expressed as a function of onset of blood lactate accumulation (4 mmol·L^−1^) values, results showed β-alanine supplementation significantly increased heart rate and %VO_2max_. In contrast placebo administration elicited non-significant changes for those same variables. However β-alanine-dosed subjects also had a significant decline in their VO_2max_ expressed in both absolute and relative terms [56].

Additional studies also assessed the presence of an ergogenic effect from oral β-alanine ingestion. With a matched-pairs design and no crossover, men received either an oral placebo or β-alanine treatment [44]. β-Alanine subjects ingested 4 g·day^−1^ for one week, followed by 6 g·day^−1^ for the next 28 days. Post-intervention both groups performed a repetitive sprint protocol. As compared to placebo data, β-alanine did not offer an ergogenic effect [44]. A similar lack of improvement to repeated sprint performance occurred at even higher (6.4 g·day^–1^) β-alanine doses given over the same time period [57]. It was implied β-alanine did not have an ergogenic effect and that muscle buffer capacity is not truly tested from repetitive sprints of short (15 m) duration [57]. In like fashion, with β-alanine administered at a rate of 4–6 g·day^−1^ for five weeks as part of double-blind placebo-controlled design, supramaximal sprints (115 and 140% VO_2max_) performed to exhaustion did not receive an ergogenic effect from supplementation [58]. Blood lactate levels, collected before and after the sprints, showed non-significant inter-group differences [58]. With similar dosing protocols and study design, β-alanine also did not increase VO_2_ max [44,56,59]. However, another trial examined 28 days of oral β-alanine (6.4 g·day^−1^ for the first six days, then 3.2 g·day^−1^ for 22 days) on cycling performance with decidedly different results [60]. Before and after the intervention subjects performed an incremental cycle ergometry protocol to volitional failure. Versus placebo data, β-alanine improved performance [60]. With a similar exercise protocol, like results were seen with even smaller (1.6 g·day^−1^) dosages given over the same time period [49]. The latter studies concluded β-alanine delays H^+^-induced neuromuscular fatigue [49,60].

Another physical activity mode characterized by neuromuscular fatigue from H^+^ accrual is resistive exercise. To examine the presence of an ergogenic effect, after performance of a knee extensor resistive exercise protocol, subjects were divided into a placebo or β-alanine (1.2 g·day^−1^ for 30 days) group with no crossover [61]. Both groups refrained from physical activity for 30 days, and thereafter repeated the exercise protocol. As compared to pre-treatment values, the interventions yielded no inter-group differences [61]. Yet another resistive exercise study, which used a similar intervention period with a higher (4.8 g·day^−1^) dosage, noted a different outcome [30]. With a crossover design, strength-trained men underwent separate 30-day β-alanine and placebo treatments [30]. After each 30-day period, subjects performed a six-set 12-repetition squat workout with 90-s rest periods against 70% of their 1 RM load. *Versus* the placebo condition, β-alanine significantly raised mean power values and was thus deemed to have an ergogenic effect on resistive exercise performance [30].

Since β-alanine generally had an ergogenic impact on anaerobic [30,49,60], but not aerobic [44,59], performance, research also assessed protocols that entailed major contributions from both oxidative and glycolytic metabolism, as some forms of exercise make substantial demands on both energy systems. β-Alanine (2–4 g·day^−1^) and placebo treatments were compared as part of a double-blind design with no crossover in cyclists [62]. Before and after the eight-week trial, subjects performed a simulated 100-min race on a cycle ergometer [62]. Results showed, as the race concluded with a 30-s sprint, β-alanine significantly raised peak and mean power by 11.4% and 5.0% respectively *versus* pre-treatment values. In a double-blind study with no crossover, rowers received a placebo or β-alanine (5 g·day^−1^) capsule dosing treatment [12]. Before and after the 49-day intervention they performed a 2000 m rowing ergometer test [12]. Pre-post changes showed the β-alanine group covered the distance 2.7 s faster, while placebo-dosed subjects were 1.8 s slower. Results also showed a significant correlation between intramuscular carnosine and rowing performance [12].

Other physical activity paradigms were also examined. They include repetitive bouts of exercise done in succession, with little or no rest between successive stages whereby performance is compromised by neuromuscular fatigue. A four-week double-blind study design with no crossover compared β-alanine (2.4–4.8 g·day^−1^) and placebo treatments given to 400 m sprinters [20]. Subjects performed a five-set 30-repetition isokinetic knee extensor test before and after the intervention. It was shown β-alanine improved knee extensor performance over the last two sets and thus may abate fatigue [20]. β-Alanine and placebo treatments were also compared to note their impact on incremental cycle ergometry exercise [6,63]. When β-alanine was ingested for both 28 (3.2–6.4 g·day^−1^) and 90 (2.4 g·day^−1^) days it yielded significant performance gains [6,63]. β-Alanine produced significant gains in ventilatory threshold, physical work capacity to fatigue threshold and time to exhaustion [63]. In contrast placebo-dosed subjects incurred minor changes to the same measures. For the 90-day intervention, pre-post changes entailed ~29% performance gain from β-alanine while the placebo led to negligible changes [6].

β-Alanine supplementation may elevate muscle carnosine [25]; yet its impact as an ergogenic aid is varied. β-Alanine has not improved muscle strength or VO_2max_ [30,44,51,56,59], yet it raised anaerobic thresholds and delayed fatigue with incremental exercise [6,63]. Carnosine raises exercise performance via greater muscle buffer capacity [8,12], heightened Ca^2^^+^ release [64], improved troponin C sensitivity for Ca^2^^+^ [65], reduced reactive O_2_ species accrual [66], and vasodilation [67]. There may be several reasons why some studies [9,30,50,60], but not others [8,44,61,68], saw an ergogenic effect. Many trials, due to the mode of physical activity and the type of outcome measured, offered limited insights on β-alanine’s efficacy as an ergogenic aid [1,8,30,44,53,57,59,68]. β-Alanine appears to be efficacious for brief supramaximal exercise immediately preceded by a fatiguing bout of physical activity [62]. Such paradigms evoke sharp intracellular pH losses and thus may benefit from β-alanine supplementation.

Questions about the efficacy of oral β-alanine as an ergogenic aid will persist until the merits of this dietary supplement are objectively and accurately quantified. Until it is, misconceptions about this dietary supplement and its usage will continue. While plausible to help address this issue, muscle buffer capacity measurements are somewhat limited by titration techniques and the feasibility of data collected from exercise [11,33,56]. To avoid misconceptions, new β-alanine research should now focus on simpler measurements, obtained before and after exercise, in order to best assess its ergogenic properties. Since carnosine is most active as a buffer when intracellular H^+^ accrual rates are highest [1,4], exercise that entails multiple supramaximal bouts of activity separated by short rest periods afford the best opportunity to examine β-alanine’s ability to buffer the added proton load. Normally double-blind placebo-controlled within-subject studies, whereby each participant receives multiple treatments and/or dosages, would serve as an ideal study design. However since β-alanine trials are complicated by the very stable nature of intracellular carnosine levels that in turn demand very long (>15 weeks) washout periods to limit carryover effects [12,19], matched-pairs designs, whereby subjects receive only one treatment, are preferable to within-subject studies.

Since lactate increases coincide with higher H^+^ concentrations [40,56], measurements of blood lactate levels over time likely afford the best opportunity to objectively and accurately quantify the merits of β-alanine ingestion, and are superior to prior studies that examined if supplementation merely conferred an ergogenic effect. Blood lactate kinetic investigations have historically entailed a pre- and multiple post-exercise measurements; however such studies included invasive procedures that were uncomfortable to subjects which likely compromised their exercise performance [69,70,71]. In contrast to the invasive measurements [69,70,71], the far simpler and currently popular blood lactate collection from finger tips may do as good a job quantifying the metabolite’s accrual into, and clearance from, the vasculature over time to offer an indirect assessment of buffer capacity. Prior blood lactate kinetic studies entailed supramaximal exercise, the very type of physical activity that evokes high lactate and H^+^ increases [69,70,71]. In theory β-alanine supplementation permits higher work capacities and yield greater peak blood lactate concentrations, due to increased proton buffering. This theory concurs [4,72,73,74] and is in contrast to [1] text from prior reviews that examined the workload-lactate relationship. As assessed through blood lactate kinetics, higher intracellular lactate accrual should also result in a faster rate of entry into the vasculature. It may be hypothesized that β-alanine supplementation will improve intracellular H^+ ^buffer capacity and permit greater exercise performance as higher amounts of lactate accrue within, and are removed from, the engaged musculature.

## 3. Recommended β-Alanine Dosages

Just like any dietary supplement, optimal β-alanine dosages are based on factors such as a person’s age, gender and nutritional/health practices. Unfortunately most studies have used young- or college-age men. Results suggest [9,30,44,50,60,61,68] ergogenic effects are more likely at higher dosages. In contrast upon cessation of β-alanine intake carnosine levels decline in a linear fashion at a relatively slow rate of 2%·week^−1^ until pre-supplementation values of the dipeptide are restored [11]. High initial intramuscular carnosine levels do not apparently limit a cell’s ability to accrue further increases of the dipeptide [20]. Some believe persons with naturally lower absolute carnosine concentrations (those with high percentages of type I muscle fibers, women, vegetarians, elderly) respond best to oral β-alanine ingestion [11,52]. A single 400 mg β-alanine dose produced favorable changes in blood-based markers, but did not improve exercise performance as compared to a placebo intervention [8]. Such a dose might represent first pass liver metabolism that must be overcome before deposition into muscle cells occurs. For 30-day interventions devoid of physical activity a 4.8 g·day^−1^, but not a 1.2 g·day^−1^, β-alanine dosage had an ergogenic effect on resistive exercise performance [30,61]. Thus the minimal β-alanine dose needed for an ergogenic effect may reside between 1.2 and 4.8 g·day^−1^ in healthy men. Dosages higher than 6.4 g·day^−1^ have yet to be studied.

## 4. Precautions

Just like any dietary supplement, β-alanine may be abused if not consumed in a proper fashion; in addition the manufacturers do not typically specify the production purity and presence of trace contaminants, which has received little prior attention [52]. β-Alanine precautions relate to its potential to induce paraesthesia, characterized by heightened sensitivity of nociceptive neurons that transmit neuropathic pain, which lead to flushing and prickly sensations on the skin [4,25,75]. The severity of paraesthesia episodes is dose-dependent but generally last 60 min after ingestion [25]. In some cases β-alanine intake was curtailed or terminated due to the severity of the paraesthesia [25]. To detect paraesthesia from acute β-alanine ingestion, six men received a series of 10–40 mg·kg^−1^ body mass dosages [25]. Acute ingestion evoked mild flushing at the 10 mg·kg^−1^ body mass dose, while significant paraestheisa occurred at 20 mg·kg^−1^ body mass. Men who experienced mild flushing at the 10 mg·kg^−1^ body mass dose did so within 20 min of ingestion [25]. When β-alanine was consumed as part of a chicken broth elixir at the 40 mg·kg^−1^ body mass dose no side effects occurred, yet when that same dosage was given as an oral dietary supplement some subjects incurred paraesthesia [25]. Given this finding, it appears in addition to the peak dosage given, the occurrence of β-alanine-induced paraesthesia episodes may relate to the manner of its administration. It was suggested individual β-alanine doses should mimic those incurred from a normal diet to limit the risk of paraesthesia [25].

The same project also assessed chronic β-alanine administration in healthy men [25]. Several dosing strategies were examined; one of which entailed intake of a 10 mg·kg^−1^ body mass dose three times per day for two weeks [25]. The dosing protocol elicited few side effects that included occasional mild flushing [25]. Two other strategies entailed 3.2 and 6.4 g·day^−1^ given to healthy men for four weeks in 400–800 mg doses [25]. The four-week protocols produced a few cases of mild paraesthesia and a sore throat in one subject [23]. Thus it appears paraesthesia-like symptoms begin at dosages above 10 mg·kg^−1^ body mass. The prevalence and severity of paraesthesia relate to peak β-alanine levels in the bloodstream which led to the demand for time-release versions of the supplement.

Plasma kinetics and occurrence of paraesthesia-like symptoms were monitored as part of a single-blind randomized trial that included examination of a time-release β-alanine supplement [76]. Healthy adults (*n* = 11) received three treatments: 1.6 g of oral time-release β-alanine, an equal single supplement dose administered in customary (bolus) fashion, and a placebo. For six hours after administration of each treatment urinary-, plasma- and questionnaire-based outcomes were obtained from subjects. Results showed the time-release version of β-alanine produced smaller peak plasma levels of the amino acid, as well as a longer lag time until peak values occurred [76]. The time-release product had less urinary loss, which may denote more β-alanine retention. The single bolus administration caused paraesthesia-like symptoms, yet the time-release version of the supplement and the placebo did not differ with respect to adverse effects. It was concluded the time-release version of β-alanine, with its lower peak plasma values, mean larger absolute doses can be consumed without the likelihood of paraesthesia [76]. While no β-alanine studies have been conducted with persons predisposed to paraesthesia, it may prudent for such people to refrain from this dietary supplement.

## 5. Implications/Future Recommendations

Despite recent studies, unanswered questions still remain. While numerous trials examined its role as an ergogenic aid, the potential of oral β-alanine intake to improve buffer capacity has yet to be fully quantified. It is thus important to measure, from both *in-vitro* and *in-vivo* trials, for a given oral β-alanine dosage the (1) amount of carnosine accretion, and (2) magnitude of increase in intracellular buffering capacity. Since lactate offers an indication of the degree of H^+^ accrual and acidosis [40,56], measurements of blood lactate concentrations over time foretells concurrent intracellular changes in response to periods of exercise and recovery. Thus in contrast to prior reviews [1,2,3,4,5], in order to quantify the efficacy of oral β-alanine ingestion, multiple blood lactate measurements before and after exercise may best address this issue and represent the primary future recommendation of the current paper. Such investigations represent a true step forward in the understanding of the ergogenic properties of β-alanine. In theory β-alanine intake should permit higher work capacities before acidosis-induced exercise cessation, despite attainment of greater peak blood lactate levels [4,74]. New trials should compare changes to blood lactate values from supramaximal exercise bouts performed with and without prior β-alanine supplementation. Until such studies are conducted on physical activity paradigms that entail successive bouts of supramaximal exercise, the true extent of ergogenic effects from β-alanine will remain unresolved. Future recommendations also include determination of optimal β-alanine doses for persons based upon their age, gender and nutritional/health practices. The occurrence of paraestheia in a variety of human subjects, from both standard and time-released versions of the supplement, should continue to be assessed [76]. Finally we recommend research into the potential toxicity of β-alanine with long-term (>12 weeks) use.
